# Histological characteristics of hair follicles at different hair cycle and *in vitro* modeling of hair follicle-associated cells of yak (*Bos grunniens*)

**DOI:** 10.3389/fvets.2023.1277586

**Published:** 2023-11-17

**Authors:** Bo Liao, Yan Cui, Sijiu Yu, Junfeng He, Xue Yang, Shengnan Zou, Sijie Li, Pengfei Zhao, Hongwei Xu, Min Long, Xiaoyan Wang

**Affiliations:** ^1^College of Veterinary Medicine, Gansu Agricultural University, Lanzhou, China; ^2^Gansu Province Livestock Embryo Engineering Research Center, Lanzhou, China

**Keywords:** yak, hair cycle, fibroblasts, preadipocytes, induced differentiation, dermal papilla cells, microdissection

## Abstract

To adapt to the extreme conditions of plateau environments, yaks have evolved thick hair, making them an ideal model for investigating the mechanisms involved in hair growth. We can gain valuable insights into how hair follicles develop and their cyclic growth in challenging environments by studying yaks. However, the lack of essential data on yak hair follicle histology and the absence of *in vitro* cell models for hair follicles serve as a limitation to such research objectives. In this study, we investigated the structure of skin tissue during different hair follicle cycles using the yak model. Additionally, we successfully established *in vitro* models of hair follicle-associated cells derived from yak skin, including dermal papilla cells (DPCs), preadipocytes, and fibroblasts. We optimized the microdissection technique for DPCs culture by simplifying the procedure and reducing the time required. Furthermore, we improved the methodology used to differentiate yak preadipocytes into mature adipocytes, thus increasing the differentiation efficiency. The introduction of yak as a natural model provides valuable research resources for exploring the mechanisms of hair growth and contributes to a deeper understanding of hair follicle biology and the development of regenerative medicine strategies.

## 1 Introduction

The yak, also known as the “ship of the highlands,” has evolved through the process of natural selection and adaptation to become the only bovine species capable of thriving in the unique ecological conditions of the plateau (1). To withstand the harsh plateau environment, the yak has developed a dense fur coat, which offers protection against extreme cold, intense ultraviolet radiation, and microbial invasion. The hair follicle, as a crucial component of the skin, undergoes cyclic changes consisting of anagen, catagen, and telogen phases, allowing periodic growth and shedding of hair (2). The proportion of time occupied by the anagen phase influences the length and growth rate of hair in different regions of the body, such as the eyebrows (3, 4), eyelashes (5), and the hair (6). Previous studies have explored the morphological characteristics of hair follicles at different stages in species such as mice (7, 8), camels (9), humans (10, 11), and goats (12). However, to date, there have been no reports evaluating the morphological structure of hair follicles during different cycles in yaks. With the rapid advancement of biological data-omics, ample opportunities for scientific research have arisen; however, stricter and more precise sample collection requirements are necessary. Failure to accurately evaluate histology can lead to errors during the periodic assessment of the collected skin samples. Thus, this study used histological techniques to comprehensively characterize hair follicles in different stages, aiming to serve as a reference to determine the growth cycle of yak hair follicles.

In addition, there is still a lack of *in vitro* models for hair follicle-related cells, particularly in yak hair follicle research. Currently, verification methods to explore the mechanisms of yak hair follicle growth rely mainly on *in situ* tissue analysis, lacking confirmation at the cellular level (13, 14). Therefore, to gain a deeper understanding of the mechanisms involved in yak hair follicle growth, it is essential to develop a suitable *in vitro* model at the cellular level. In this study, we identified the optimal culture conditions for primary cells that are suitable for the investigation of yak hair follicles. Such an *in vitro* model will provide a more direct, controllable and observable system for investigating the growth, differentiation, and regulatory mechanisms of yak hair growth mechanisms.

Within the regulation of important transitions and regeneration of the hair follicle period, basal dermal papilla cells (DPCs) play a pivotal role in orchestrating cascades of signaling interactions with surrounding cells and the epithelial matrix (7, 15–19). These intricate signaling networks guide the proliferation, migration, differentiation, and apoptosis of various cell types that constitute the hair follicle, thus facilitating its cycling and regeneration. Transplantation experiments of DPCs have demonstrated their potential for hair follicle regeneration in mice and humans (20, 21), providing novel strategies in this field. However, efficient *in vitro* culture of DPCs poses several challenges, including low isolation and culture efficiency, as well as loss of biological characteristics after prolonged periods of *ex vivo* cultivation (21, 22). Recently, significant advances have been made in maintaining the long-term inductive properties of DPCs cells through the implementation of strategies utilizing three-dimensional (3D) culture systems (21, 23, 24) and co-culturing techniques involving keratinocytes (25). Therefore, the development of effective DPCs culture protocols allowing large-scale expansion remains a significant bottleneck in research on hair follicle regeneration.

Fibroblasts are one of the most abundant cell populations in the dermis, but their function is often underestimated. Heterogeneous fibroblasts are commonly classified into two lineages, papillary and reticular, according to their location (25). During wound healing, papillary fibroblasts can induce the regeneration of hair follicles and other appendages (26). In contrast, reticular fibroblasts tend to secrete large amounts of extracellular matrix (ECM), leading to scar formation and lack of original appendages (27, 28). However, altering signaling pathways in reticular fibroblasts can achieve hair follicle regeneration at wound sites (29–31), indicating the plasticity of fibroblasts. Furthermore, fibroblast-induced hair follicle regeneration can also occur through exogenous chemical induction (32) or biotin treatment (33). In-depth understanding and research of fibroblasts holds great promise for applications in hair follicle regeneration, skin tissue engineering, and other fields.

Within the adipose lineage, cells in different stages exert intricate effects on hair follicle growth. Adipose-derived stem cells (ADSCs) and preadipocytes have been shown to exert a promotive effect on hair follicle growth. *In vitro* studies have shown their ability to enhance dermal papilla cell proliferation (34). Similarly, *in vivo* administration of ADSCs (35), their conditioned medium (36) or exosomes (37) by intradermal injection has produced positive results in terms of improving the density of the primary hair follicle and improving skin quality. In contrast, mature adipocytes negatively affect hair follicle growth. Co-culture of hair follicle cells with mature adipocytes *in vitro* triggers follicular cell apoptosis (38). Furthermore, when hair follicles infiltrate subcutaneous adipose tissue *in vivo*, they undergo a rapid catagen transition and regression (39). Obesity further disrupts the Sonic hedgehog (SHH) signaling pathway in hair follicle stem cells, resulting in aberrant differentiation, depletion, and subsequent hair follicle atrophy and alopecia (40).

Interestingly, seasonal changes in fat deposition (41) and the hair cycle (42) have also been observed in yaks under natural grazing conditions. This suggests that, in addition to environmental factors, there may be regulatory effects between yak adipocytes and hair follicles, but cell-level validation is still lacking. *In vitro*, mature adipocytes are typically obtained by culturing preadipocytes or adipose-derived stem cells and inducing their differentiation into adipocytes. Although the methods for the culture of preadipocytes show general similarities between species, there are significant differences in the induction of mature adipocytes (8, 43). Furthermore, existing induction protocols present drawbacks, such as low efficiency and high time consumption. Therefore, in this study, while cultivating yak preadipocyte models, we evaluated the effectiveness of three induction methods to differentiate yak preadipocytes into mature adipocytes. Our aim was to establish stable *in vitro* models of two different stages of adipocytes in a shorter time, providing an efficient and stable cell model to explore regulatory mechanisms and effects of the two adipocyte lineages on the growth of the yak hair follicle.

In conclusion, as an excellent model for studying hair regeneration in a high-altitude environment, yaks still require accurate histological identification criteria for hair follicle structure and an *in vitro* cell model that can be used to study interactions between hair follicle cells. Establishing these *in vitro* cell models can also be used to simulate mechanisms under external conditions specific to high-altitude environments, such as hypoxia, ultraviolet radiation, and low temperature. This is of great significance to fully understand the adaptive mechanisms of yaks in high-altitude environments.

## 2 Materials and methods

### 2.1 Experimental animals

In this study, 15 yaks were obtained from Xining City in Qinghai Province, China. According to the division time of the yak hair follicle cycle established by Zhang et al. (9) and Yang et al. (10), skin samples of anagen, catagen, and telogen were collected from 9 of the yaks (male, *n* = 6; female, *n* = 3). Cultures of fibroblasts, preadipocytes, and dermal papilla cells were derived from yaks older than 1.5 years (male, *n* = 3, female, *n* = 3). All experimental yaks were in good nutritional condition. The animal care and experimental protocols were approved by the Animal Ethics Committee of Gansu Agricultural University and conducted according to the Animal Ethics Regulations of the People’s Republic of China.

### 2.2 Primary cell cultures

#### 2.2.1 Primary fibroblast culture

Yak dermal fibroblasts were cultured according to the methods described by Khan et al. (11). Under local anesthesia, abdominal skin specimens (1–1.5 cm^2^) were collected. The skin was washed with 70% alcohol and physiological saline solution containing antibiotic-antifungal solution (5,000 U/mL penicillin, 5,000 μg/mL streptomycin, 20 μg/mL amphotericin B) before being transported to the laboratory. The dermal tissue of the yak skin was separated using sterile tweezers under laminar flow conditions (Supplementary Figure S1), followed by washing with a phosphate-buffer solution (PBS) (pH = 7.2) (pH = 7.2) containing antibiotic-antifungal agents. Dermal tissue was isolated and cut into 1 mm^3^ blocks before being transferred to 25cm^2^ cell culture flasks and incubated in a humidified atmosphere of 5% CO_2_ at 37°C for 2–4 h until the explants adhered to the bottom of the plates. Subsequently, DMEM/F-12 medium supplemented with 10% FBS and 1% antibiotic-antifungal solution was added in a 5 mL volume, which was replaced every 2 days until cells reached confluency levels between 80 and 90%.

#### 2.2.2 Primary culture of preadipocytes

We employed an enzymatic digestion method modified by Yang et al. (12) to cultivate preadipocytes. Briefly, after euthanizing two yaks (the experimental yaks were euthanized for tissue sampling for parallel studies by our research group), subcutaneous adipose tissue was obtained from the groin area and rinsed with a normal saline solution containing antibiotic-antifungal solution (5,000 U/mL penicillin, 5,000 μg/mL of streptomycin; 20 μg/mL of amphotericin B). The collected adipose tissue was then cut into 1–3 mm^3^ pieces, thoroughly washed and subjected to enzyme digestion by adding 4X collagenase I enzyme to a 50 mL centrifuge tube. The mixture was incubated at 37°C for 2 h on a shaker and then filtered through DMEM/F-12 medium containing 10% FBS. The cell precipitate was resuspended by centrifugation at 1500 × g for 10 min, repeating twice. The resuspended cells were then seeded in 25 cm^2^ cell culture flasks.

#### 2.2.3 Primary culture of dermal papilla cells

Yak hair follicle dermal papilla cells were cultured as described below using the methods described by Gledhill et al. (44), Topouzi et al. (45), and Limbu et al. (46), with modifications:

1. As previously described in Section 2.2.1, a total of approximately 1.5 cm^2^ of abdominal skin tissue was collected from the yaks, followed by washing with 70% alcohol and physiological saline solution containing antibacterial and antifungal agents before transporting the samples to the laboratory.
2. Subsequently, the skin tissue was transferred to 100 mm^2^ cell culture plates and longitudinally sliced. The slice thickness was approximately 0.1 cm, which strikes a balance between maintaining the amount of hair follicles and facilitating their separation.
3. Blunt dissection was used to remove the dermis and subcutaneous tissue beneath the hair bulb, while simultaneously maximizing the exposure of the hair bulb (Supplementary Figure S1).
4. The skin tissue containing hair follicles was washed three times with DMEM/F-12 supplemented with antibiotic-antifungal solution before being transferred to a new 35 cm^2^ cell culture plate. Subsequently, fine tip tweezers (RWD, F11002-11) and spring scissors (RWD, S11007-12) were used to selectively separate hair bulbs from hair follicles.
5. The collected hair bulb tissues were placed in a new 35 mm dish, supplemented with DMEM/F-12 containing 2% collagenase IV, and smoothly transferred to an incubator at 37°C for 2 h.
6. When reaching this stage, the tissue architecture surrounding the outer root sheath of the hair follicle had achieved sufficient laxity, which enabled the easy extraction of the dermal papilla of the hair follicle from the base of the hair bulb using a fine needle (27G x3/4″).
7. Eight independent dermal papillae were meticulously isolated and transferred to individual wells in a 24-well plate at a density of eight papillae per well. Subsequently, 1 mL of DMEM/F12 medium was added to each well. The medium was replaced after observing successful migration of cells from the papillary cultures.

### 2.3 Cell immunofluorescence and cell growth curve

Cultured cells were fixed for 15 min in 4% paraformaldehyde, permeabilized with 0.1% Triton X-100 for 30 min, then subsequently blocked for 1 h with 1% BSA and incubated with the primary antibody for 1 h. The primary antibodies used in the experiment were anti-vimentin (VIM) (dilution: 1:500; abcam, ab8069) and anti-α-SMA (dilution: 1:200; Beyotime, AF1507). After washing with neutral PBS three times, cells were incubated with secondary antibodies in the dark for 1 h. Cell nuclei were stained with DAPI for 3 min. The slides were then sealed with neutral resin.

Cell growth curves were determined following the manufacturer’s protocol for the CCK-8 assay kit (Bioss, BA00208). Briefly, yak dermal fibroblasts, preadipocytes, and dermal papilla cells from the third passage were seeded in 96-well plates at a density of 2000 cells per well (7 groups total, 4 wells per group). Groups were tested every 24 h, 10 μL of the WST-8 solution of the CCK8 kit was added to each well and incubated for 2 h under 5% CO2 at 37°C. The WST-8-containing medium was then transferred to new 96-well plates and the absorbance at 450 nm was measured using a microplate reader (Bio-Rid, iMark 18,717). Growth curves for each cell type were generated by plotting the absorbance values of 450 nm over time.

### 2.4 Differentiation of yak preadipocytes into adipocytes

We set up three different lipid induction regimens to evaluate their efficiency in transforming yak preadipocytes into mature adipocytes. Each induction method contained a complete differentiation medium (DMa) and a maintenance medium (DMb). All basal media (BM) consisted of DMEM/F12 with 10% FBS and 1% antibiotic-antifungal solution. The complete differentiation medium and maintenance differentiation medium were formulated as follows:

- DM1a: BM, 0.5 mM isobutyl methylxanthine (IBMX) (MCE, HY-12318), 1 mM dexamethasone (MCE, HY-14648), 10 μg/mL insulin (Beyotime, P3376) and 10 μM rosiglitazone (MCE, HY-17386).
- DM1b and DM3b: BM and 10 μg/mL insulin.
- DM2a and DM2b: BM and 160 μM oleic acid (OA) (Solarbio, SC9320).
- DM3a: BM, 0.5 mM isobutyl methylxanthine (IBMX), 1 mM dexamethaone, 10 μg/mL insulin, 10 μM rosiglitazone, and 160 μM OA.

**Figure 1:**
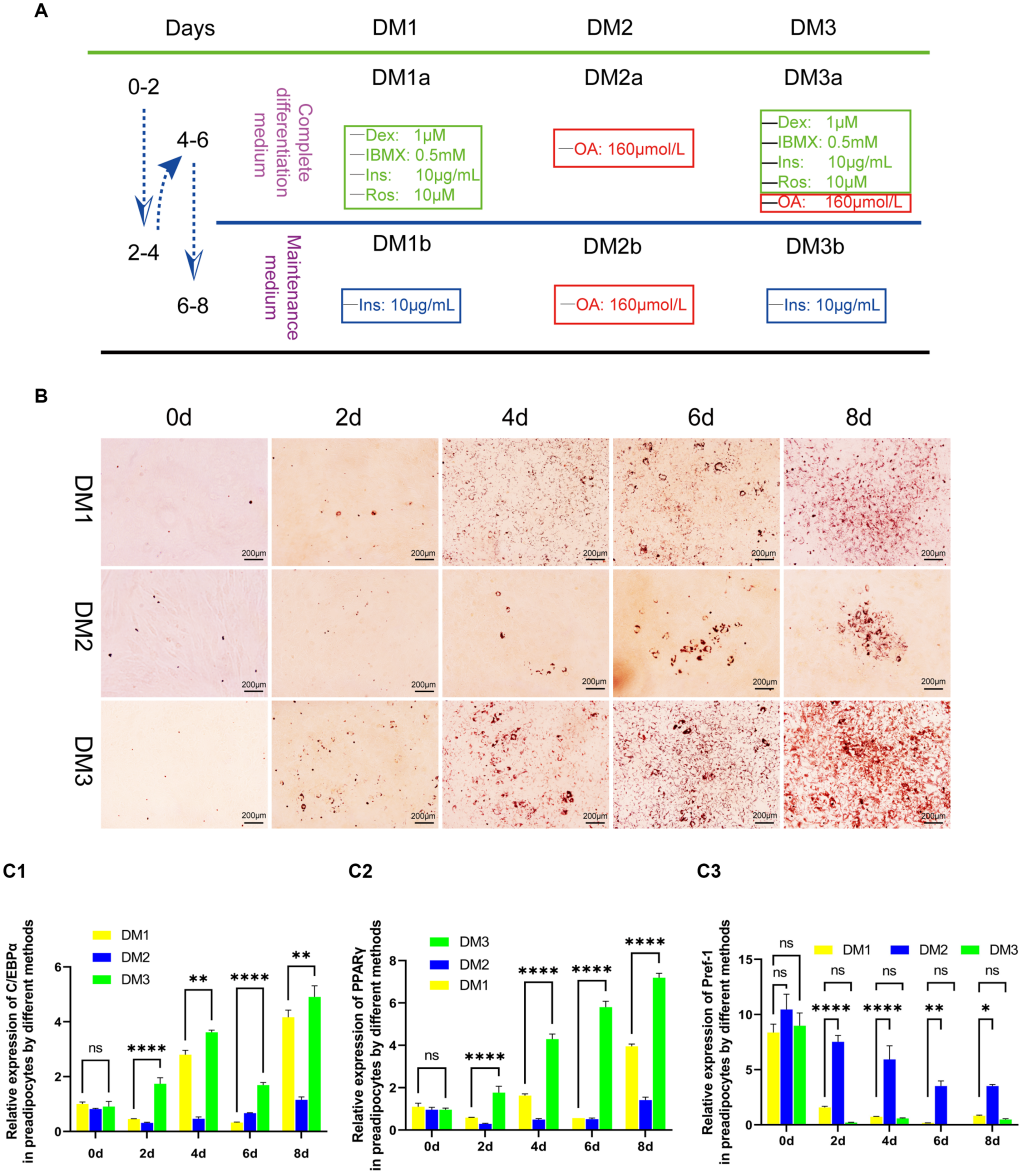
Evaluation of the transformation of yak preadipocytes into adipocytes by three induction regimens. **(A)** Introduction to induced differentiation schemes. **(B)** Adipocyte differentiation induced by three induction schemes at different time points (Oil-Red-O staining). **(C)** The expression of key genes (PPARγ, C/EBPα, and Pref-1) during the transformation of adipose precursor cells into adipocytes by three induction regimens. **B**:100 × .

Following the achievement of a 90% confluence of yak preadipocytes (P3) in a 35-mm culture plate, they were subjected to a sequential differentiation protocol. Initially, cells were exposed to complete differentiation medium (DM1-3a) for a duration of 2 days, after which they were transitioned to maintenance medium (DM1-3b) for another 2 days. This process was repeated once. Adipocytes were harvested at specific time points (0, 2, 4, 6, and 8 days) for subsequent analyzes, including Oil-Red-O staining to assess lipid accumulation and gene detection of key adipogenic transcription factors. The experimental differentiation protocol is illustrated in Figure 1A.

### 2.5 Oil red-O stain

At designated time points (0, 2, 4, 6, and 8 days) after the differentiation of the yak preadipocytes, cells were washed twice with DPBS, fixed in 10% formalin for 30 min, washed again with DPBS, and incubated with 60% isopropanol for 5 min. Subsequently, the cells were treated with a fresh oil Red-O solution (Solarbio, G1262) for 5 min at room temperature. The excess dye was removed by washing several times with DPBS, and finally bright-field images were obtained.

### 2.6 Quantitative real-time PCR

Quantitative real-time PCR was performed using ABIViiA7 (Life technologies, United States) with the Go Taq qPCR Master Mix kit (Promega, USA) in a 20 μL reaction. Using Primer 5 software, primers were designed based on the corresponding gene sequences and synthesized at Sangon Biotech Company in China (Table 1). The following temperature conditions were used for PCR: 95°C (30 s), 95°C (4 s), 60°C (1 min), and 72°C (30 s), for 42 cycles in total, with 72°C (10 min) as the final extension condition. Electrophoresis (1.5% agarose gel) of amplified PCR products was performed and quantification of relative gene expression was performed using Image-QuanT software (Molecular Dynamics, Sunnyvale, CA, United States). The comparative Ct value was calculated with β-actin as internal control. Each PCR experiment was performed in triplicate and was repeated at least three times.

**Table 1:** Primers and annealing temperature for real-time PCR.

Gene	Primer Sequence(5′-3′)	Annealing (°C)	Length (bp)
PPARγ	(F-) GACCACTCCCATGCCTTTGA	60	109
	(R-) CAACCATCGGGTCAGCTCTT		
C/EBPα	(F-) TGTCCCACGGGACCTACTAC	60	134
	(R-) GTAGGCAGACAGGTCGATGG		
Pref-1	(F-) CTCAACAAGAGCACTCCGCT	58	149
	(R-) TGGTTGTAGCGCAGATTGGA		
β-actin	(F-) AGGCTGTGCTGTCCCTGTATG	60	207
	(R-) GCTCGGCTGTGGTGGTAAA		

### 2.7 Light microscopy

The skin samples of the yaks were fixed on paperboard to prevent shrinkage, stored in a 4% paraformaldehyde solution (Solarbio, P1110), softened, dehydrated, embedded in paraffin, sectioned to a thickness of 5 μm and deparaffinized. The sections were stained using the Sacpic method (45).

### 2.8 Measurement and data analysis

To evaluate the density and activity of secondary hair follicles (SF) in our study, we used a computerized light microscope (Olympus DP71) to capture transverse sections of the samples. These images were then analyzed using morphometric software (Image-Pro plus 6.0). For each skin sample, we observed 30 groups of follicles to estimate the density of the SF. The numbers of the SF and active SF were individually counted and converted into densities per square millimeter (/mm^2^) for both categories. Active SF was identified by the presence of red IRS (inner root sheath). To determine the activity of the SF, we calculated the ratio of their numbers with red IRS to the total number of SF within each sample.

To assess the depth and diameter of the SF beneath the skin surface, we captured longitudinal sections of the samples using the same computerized light microscope (Olympus DP71) and measured them using morphometric software (Image-Pro plus 6.0). A total of 30 SF per skin sample were analyzed to estimate their depth and diameter.

All data were presented as mean ± standard deviation (SD). To analyze statistical significance, we used the Statistical Package for Social Science software, version 19.0 (SPSS Inc., Chicago, IL, United States). Our primary statistical analysis involved a one-way analysis of variance (ANOVA). A significance level of *p* < 0.05 was used to determine statistical significance.

## 3 Results

### 3.1 General structural characteristics of hair follicles

**Figure 2:**
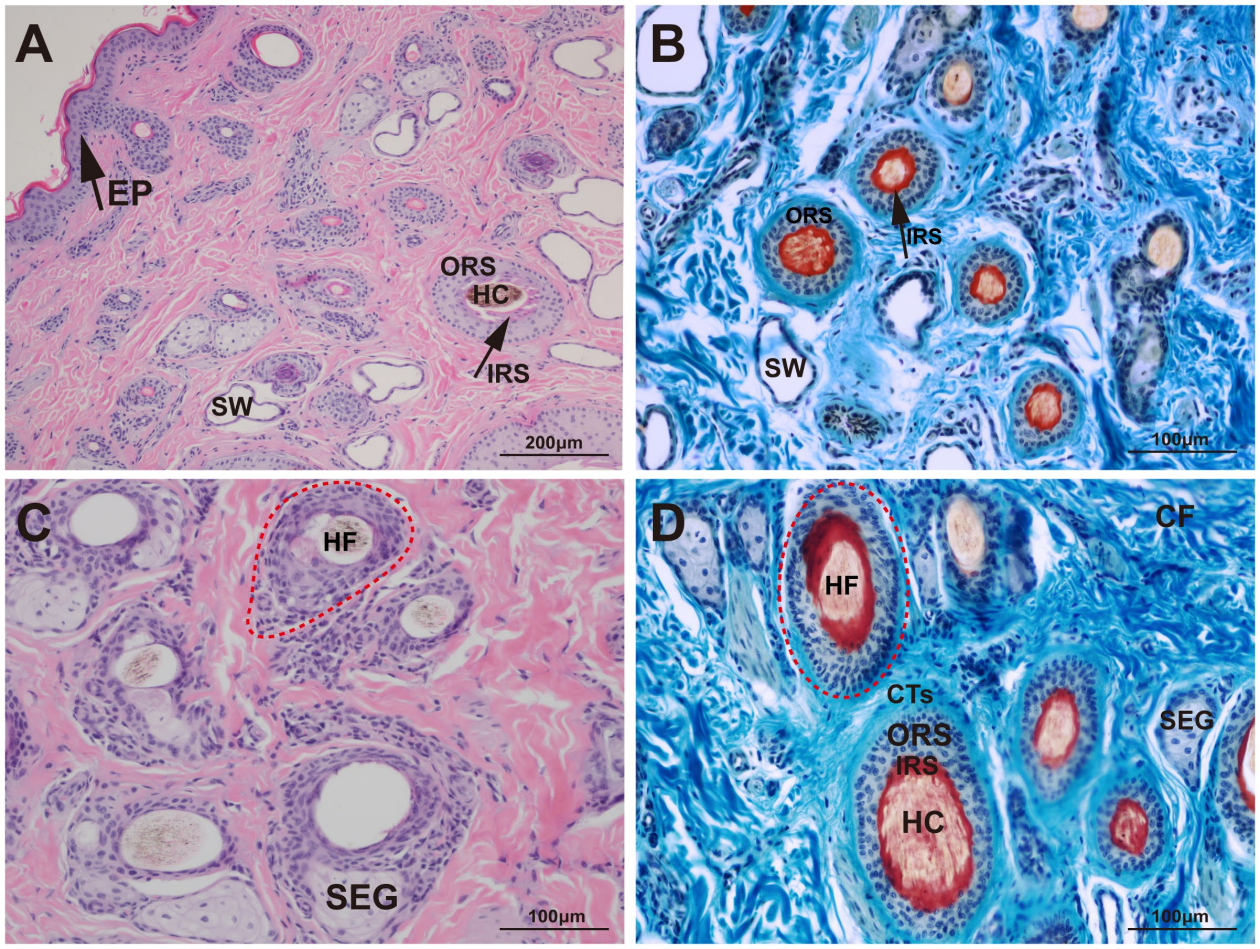
Histological structures of the hair follicle in yak. HF, hair follicle; HC, hair cortex; IRS, inner root sheath; ORS, outer root sheath; CTs, connective tissue sheath; SW, sweat gland; SEG, sebaceous gland; CF, collagen fiber; EP, epidermis. **(A)** HE, 100×; **(B)** Sacpic, 200×; **(C)** HE, 200×; **(D)** Sacpic, 200 × .

The structural characteristics of the yak skin were visualized using Sacpic staining, in which pigment particles were stained black, collagen fibers blue, smooth muscle fibers green, inner root sheath bright red, outer root sheath gray-green, connective tissue sheath blue-green, nucleus blue-purple, and keratin yellow. Microscopic observation revealed hair follicles were arranged in groups and enclosed by a connective tissue sheath (Figure 2).

### 3.2 Structural characteristics of hair follicle groups in different stages of the hair cycle

**Figure 3:**
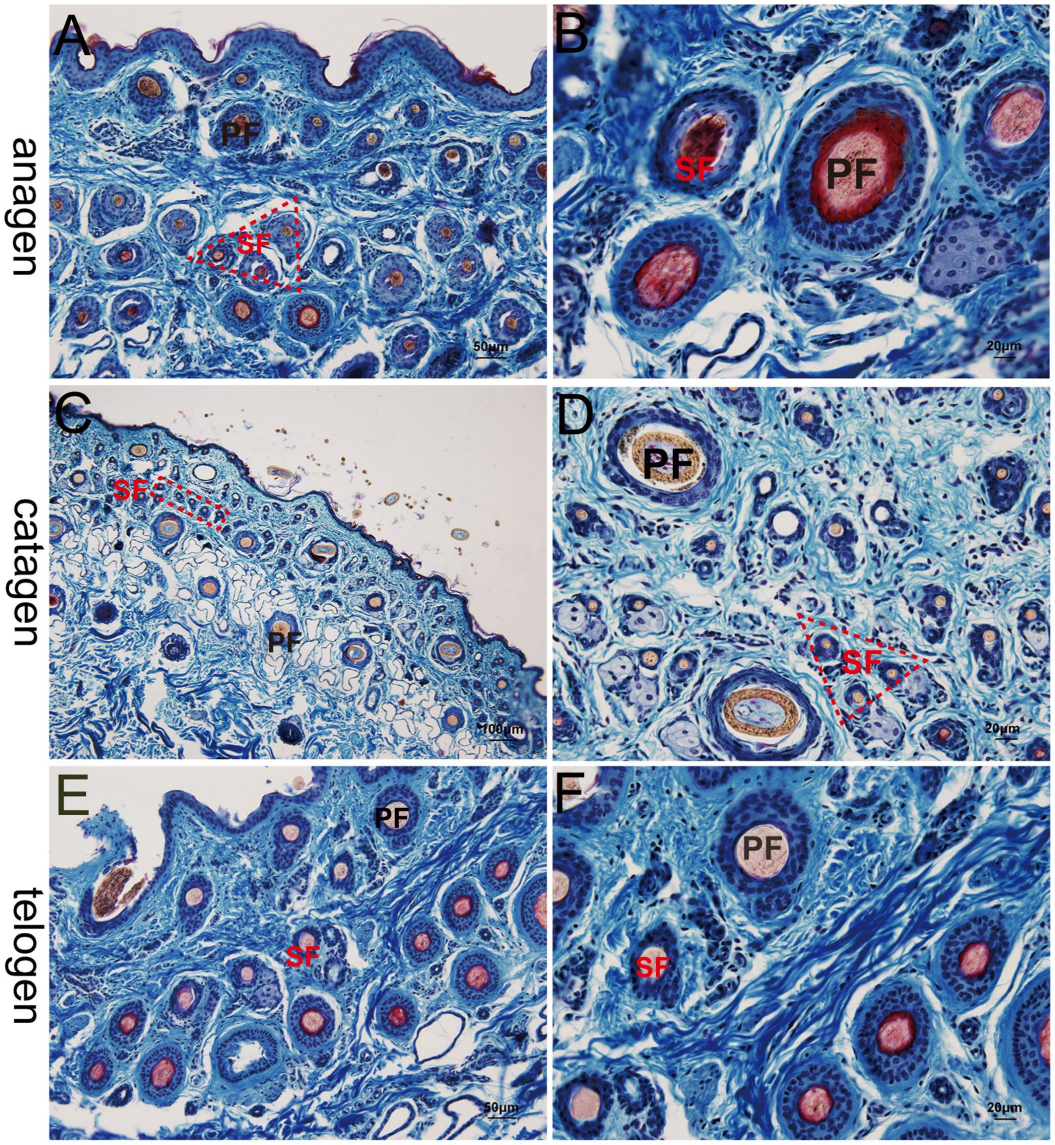
Histological structures of different hair follicle cycles in yak, Sacpic. PF, primary follicle; SF, secondary follicle; IRS, inner root sheath; ORS, outer root sheath. **(A,B)**: anagen phase; **(C,D)** catagen; **(E,F)**: telogen; **(A,E)**: 200×; **(B,D,F)**: 400×; **(C)**: 100 × .

At the telogen stage, the structure of the hair follicle group was observed to be loose. The connective tissue sheath and the outer root sheath of most primary hair follicles were thin, and a bright red inner root sheath was absent. Some hair shafts had fallen, leaving empty cavities enveloped by the outer root sheath. The SF were small and mostly dark clumps (Figures 3E,F). Upon entering the anagen stage, the structure of the hair follicle group became more evident and complete, with significantly more primary and SF. The connective tissue sheath and the outer root sheath of the primary follicles thickened and densified. Hair stems developed and the SF exhibited the inner and outer root sheath. The structure of the inner root sheath gradually became complete (Figures 3A,B). As the hair follicle group entered the catagen stage, the number of primary and SF began to decrease, some primary follicles lost the medulla, the hair shaft began to fall out, and the SF atrophied and decreased in size. The inner red root sheath increased, becoming shorter, and the outer root sheath also became thinner (Figures 3C,D). The distinct characteristics of the follicular structures observed by Sacpic staining are summarized in Table 2.

**Table 2:** Structural features of hair follicles during the hair cycle in yak.

Stage	Medulla	Brush end	IRS	Follicle bulb	ORS	Hair germ
Telogen	A	P	A	A	P	P
Anagen	P/A	P/A	P	P	P	A
Catagen	A	P/A	P/A	P/A	P/A	P/A

Letters represent whether a feature was always present (P), always absent (A), or sometimes present (P/A).

### 3.3 Characteristics of secondary follicles in different stages of the hair cycle

Data relative to SF during different stages of the hair cycle are shown in Table 3. The depth, diameter, density, and activity of the SF in the skin varied by stage. The deepest SF were observed in the anagen stage (1294.278 ± 196.402 μm). The shallowest and smallest SF were in the telogen stage (841.314 ± 188.713 μm). The difference between the anagen stage and the other stages was significant (*p* < 0.05). The largest diameter of the SF was in the anagen stage (101.823 ± 23.826 μm). The smallest diameter of the secondary follicle was in the telogen stage (69.256 ± 24.565 μm). The diameter of the anagen was significantly greater than in other stages (*p* < 0.05). Secondary follicle density exhibited stage-dependent changes during the hair cycle. The density was lowest in the telogen stage. After entering the anagen stage, the density of the SF gradually increased. Significant differences were found between the three stages (*p* < 0.05). Secondary follicle activity was lowest in the telogen stage. Activity increased upon entry of anagen, then gradually decreased entering the catagen stage, but remained higher than that in the telogen stage. Telogen activity was significantly lower than in other stages (*p* < 0.05).

**Table 3:** Statistical analysis of SF during the hair cycle in the yak.

Stage	SF depth (μm)	SF diameter (μm)	SF density (/mm^2^)	SF activity (%)
Telogen	841.314 ± 188.713^b^	69.256 ± 24.565^b^	34.428 ± 12.875^c^	33.889 ± 23.319^b^
Anagen	1294.278 ± 196.402^a^	101.823 ± 23.826^a^	42.123 ± 13.084^b^	56.873 ± 18.608^a^
Catagen	906.319 ± 153.715^b^	89.174 ± 25.668^b^	53.616 ± 16.409^a^	51.667 ± 15.494^a^

Different letters represent that the difference was significant (*P* < 0.05), and the same letter represents that the difference was not significant (*p* > 0.05).

### 3.4 Primary fibroblast culture

#### 3.4.1 Morphological characteristics and *in vitro* growth of yak fibroblasts

**Figure 4:**
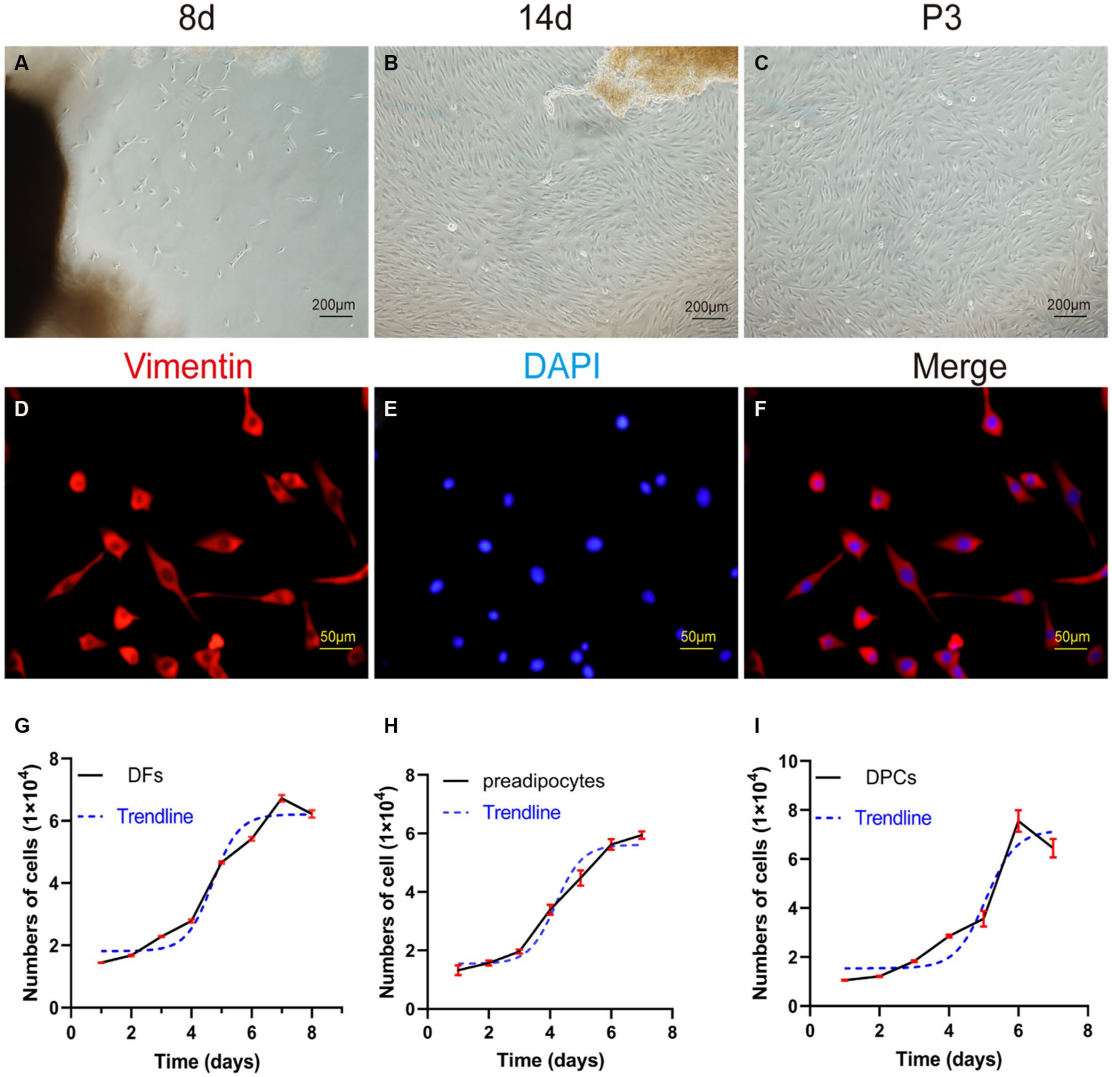
Primary culture of dermal fibroblasts (DFs) from the abdominal skin of the yak. **(A,B)** The morphological characteristics of cell growth in skin explants were evaluated after 8 and 14 days of primary culture. **(C)** Culture of DFs of P3. **(D–F)** Immunostaining was performed to evaluate the expression of the cell marker VIM and the nucleus marker DAPI in cultured fibroblast cells at P3. Growth curves of **(G)** yak dermal fibroblasts, **(H)** yak preadipocytes, and **(I)** yak dermal papillae cells (DPCs). **A–C**: 100×; **D–F**: 400 × .

Following adhesion of tissue explants to flasks for 7–8 days, small spot-like cells emerged from skin dermal tissue fragments in the periphery. These cells showed continued growth and proliferation (Figure 4A). As the culture progressed, for 10–15 days, the morphology of the cells changed to spindle-shaped, and the cells reached 80–90% confluence (Figure 4B). Upon passaging, P3 generation cells showed a prototypical spindle-like morphology, characterized by elongated bodies, while the nuclei assumed an oval shape and were placed centrally within the cytoplasm (Figure 4C).

#### 3.4.2 Cytological identification and growth curve of fibroblasts

The P3 generation of yak dermal fibroblasts was evaluated for the expression of the cell surface-specific marker VIM (Figures 4D–F), which showed a strong positivity rate of 95%. This finding aligns with expected biological characteristics of fibroblasts, affirming their distinct cellular identity.

The growth kinetics of yak dermal fibroblasts were characterized by a classic sigmoidal pattern, as illustrated in Figure 4G. After an initial lag phase of approximately 2 days, the cells entered a rapid proliferative phase, lasting 4 days, during which exponential growth was observed. Subsequently, the cells transitioned into a stationary phase, reaching a state of growth equilibrium.

### 3.5 Primary preadipocytes

#### 3.5.1 Morphological characteristics a of yak preadipocytes

**Figure 5:**
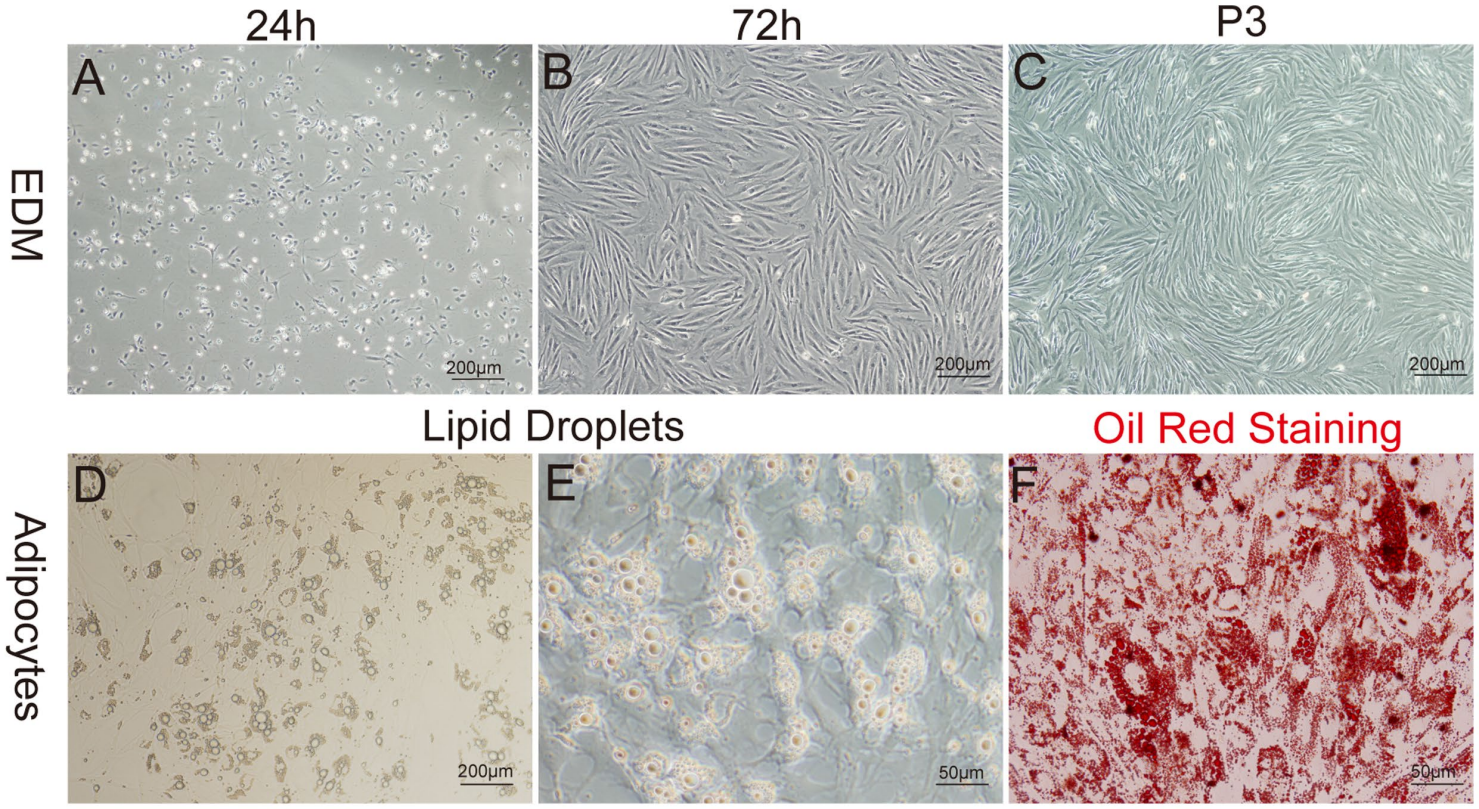
Primary culture of yak preadipocytes. **(A–C)** Morphology of yak preadipocytes cultured using the enzyme digestion method (EDM). **(D–E)** Morphological characteristics of adipocytes. **(F)** Oil-red staining of adipocytes. **A–D**:100×; **E,F**:400 × .

The enzyme-digestion method (EDM) demonstrated efficient cell attachment, resulting in a high yield of preadipocytes in a short time frame. Approximately 90% of the cells adhered to the culture substrate within 24 h of initiation. Most adhered cells initially showed a round morphology, while a subset also exhibited fusiform or irregular shapes. On day 3 of culture, a remarkable transition occurred, as more than 95% of the cells adopted a fusiform shape, further organizing into dense clusters or aggregates (Figures 5A–C).

#### 3.5.2 Growth curve and identification of preadipocytes

The growth characteristics of the third passage yak preadipocytes were evaluated by analyzing the growth curves using a previously established methodology. The growth curves of preadipocytes exhibited a typical sigmoidal pattern with distinct phases. The initial 1–3 days represented a lag phase characterized by sluggish cell proliferation. This phase was followed by a logarithmic growth phase from days 3–5, during which the cells exhibited exponential growth. Subsequently, on days 6–7, the proliferation rate of the cells began to decline, indicating the onset of a plateau phase (Figure 4H).

To identify whether the cultured cells were yak preadipocytes, we performed adipogenic differentiation experiments for validation. Specifically, following the method described by Pu et al. (47), Cells at Passage 2 (P2) were treated with DM1 adipogenic differentiation medium for 14 days. Microscopic observation and Oil-Red-O staining results (Figures 5D–F) demonstrated that abundant lipid droplets were present in the induced differentiated cells, indicating a distinct adipogenic potential of the cultured cells. This result verified that the extracted cells were indeed yak preadipocytes.

#### 3.5.3 Adipogenic differentiation

The results of induce differentiation in preadipocytes from yak adipose tissue using three different inducing agents are shown in Figure 1. Lipid droplets, stained red with Oil Red, were observed on the second day, with a progressive increase in their number and size over time. However, the effects of the three-induction media differed. Compared to the induction medium that contains only contains OA (DM2) and the traditional cocktail method (DM1), the modified cocktail method integrating both approaches (DM3) facilitated a more rapid and pronounced differentiation process of preadipocytes, as evidenced by higher differentiation scores both visually and on microscopic examination (Figure 1B).

To further assess the degree of differentiation, we used real-time fluorescence quantification to measure the gene expression levels of key transcription factors of adipocyte differentiation, namely C/EBPα and PPARγ, along with the negative regulatory factor Pref-1, which hinders adipocyte differentiation. We observed that the transcription of these three factors in DM2-induced preadipocytes showed instability with a prolonged induction period. Conversely, DM1 and DM3 initiated a gradual up-regulation of C/EBPα and PPARγ expression, accompanied by a progressive down-regulation of Pref-1 transcription. Furthermore, DM3 exhibited a faster and significantly different regulatory effect on these three factors (Figure 1C).

### 3.6 Primary culture DPCs

#### 3.6.1 Morphological characteristics a of yak DPCs

**Figure 6:**
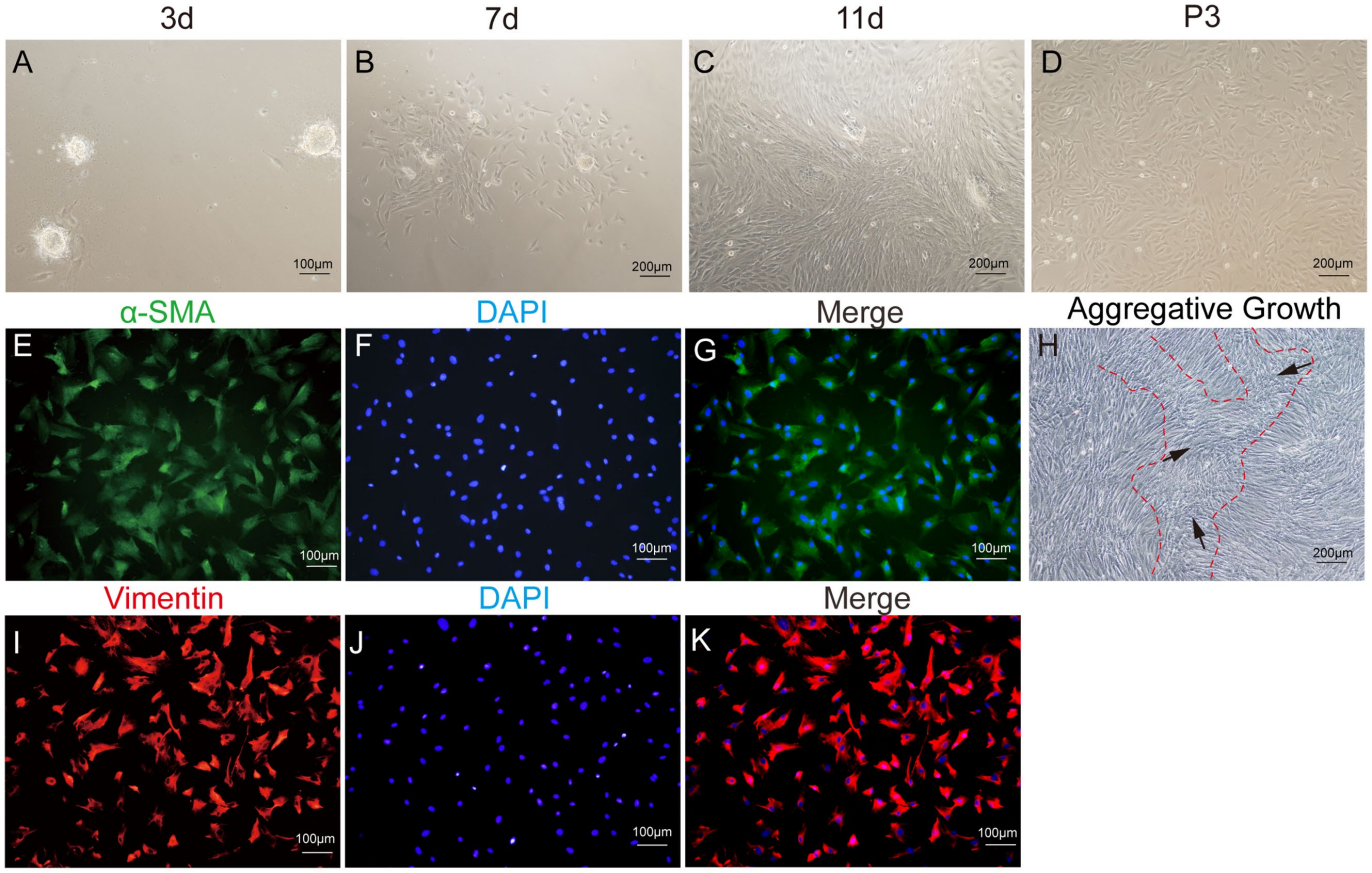
Primary culture of dermal papilla cells (DPCs) of yak. **(A–C)** Morphological observations of primary yak dermal papilla cells cultured *in vitro* on days 3, 7, and 11. **(D)** Culture of DPCs of P3. **(E–G)** Immunostaining was performed to evaluate the expression of the cell marker Vimentin (VIM) and the nuclear marker DAPI in cultured fibroblast cells at P3. **(I–K)** Immunostaining was performed to evaluate the expression of the cell marker α-SMA and the nucleus marker DAPI in cultured fibroblast cells at P3. **(H)** Aggregative growth of dermal papilla cells. **A**, **E**, **F**, and **(I–K)**, 100×; **C**, **D**, and **H**, 200 × .

After enzymatic digestion, dermal papilla explants quickly adhered to the culture surface, allowing cell migration within 1–3 days. The migrating cells presented a spindle-shaped or round-dot shape. On day 7, a significant number of cells had migrated out, adopting a spindle-shaped morphology. Subsequently, cells reached 80–90% confluence after 11–14 days of culture, and the dermal papilla gradually dissipated with the migration of dermal papilla cells (Figures 6A–D). Following the passage of cells (P3), a small fraction of cells displayed a triangular shape, while the majority assumed a fusiform (spindle-shaped) morphology. In particular, the long spindle axis of dermal papilla cells was shorter compared to fibroblasts and preadipocytes, and cells showed aggregative behavior, which was specifically manifested when cells grew close to each other and connect closely, forming cell aggregates or multilayer structures (Figure 6H).

#### 3.6.2 Detection of epithelial markers and the growth curve of preadipocytes

The third passage (P3) of yak DPCs was chosen to perform growth curve analysis and to detect cell surface markers α-SMA and VIM, following previously established protocols. The immunofluorescence assay revealed that both surface markers were positive (Figure 6). The DPCs were in the latency phase on days 1–4 and proliferated slowly. This phase was followed by a logarithmic growth phase from days 4–6, during which the cells exhibited exponential growth. Subsequently, from days 6–7, the proliferation rate of the cells started to decline, indicating the onset of a plateau phase (Figure 4I).

## 4 Discussion

### 4.1 Histological and structural characteristics of yak skin and hair follicles

In a previous study, we underscored the need to account for skin shrinkage from fixation when measuring dermal thickness (10). To mitigate this artifact, we implemented a cardboard-mounted fixation technique that permitted the acquisition of data approximating the physiological state. Additionally, Sacpic staining allowed a simple yet accurate means of determining the status of pilosebaceous unit activity (45). This approach for evaluating skin histology delineated the continuous hair cycle into active and resting phases. Under light microscopy, active phase follicles possessed a red inner root sheath that formed a stark chromatic contrast with the internal yellow hair shaft and the external gray-green outer root sheath. This conferred greater advantages in visual quantification compared to HE staining. In contrast, inactive follicles lack the red inner root sheath tissue. This facilitated quantitative analysis of active versus inactive follicle proportions in skin sections at different stages of the seasonal hair cycle, allowing the exploration of factors that modulate hair cycle and growth activity.

Nonetheless, this method has limitations. The active phase of hair follicles encompasses mainly anagen and early catagen stage follicles, while resting follicles are generally in telogen or late catagen. While classically categorizing follicles into three stages (anagen, catagen, and telogen), effective determinations can still be made by evaluating the active composite inner root sheath tissues and the global follicle cluster morphology. However, recent studies have identified a fourth phase, the exogen phase, which arises during the transition from telogen to anagen (48–50). This is characterized primarily by proteolysis at the hair base and active hair shaft shedding. Mice and humans exhibit divergent histological traits during this phase. In mice, club hair shedding coincides with the beginning of early hair growth phases, forming double-pore structures with Sacpic staining, indicating that the two phases occur concurrently, but not in identical tissue compartments (51). In humans, hair shedding can occur in the same follicle pore (16, 52, 53), meaning that a single Sacpic histological staining cannot now reliably identify this phase. Instead, accurate determination necessitates examination by immune-histochemistry and electron microscopy, which pose challenges for characterizing the exogen phase in yak as well. Although this phase has yet to garner sufficient attention, it is critical for investigating the mechanisms of hair shedding and prevention.

Table 3 shows that the depth, density and activity of the SF on yak increased after entering the anagen stage, which is consistent with previous studies on various breeds of cashmere goats (54–57). However, yaks had a lower maximum value of secondary follicle activity (56.873 ± 18.608%) than the Inner Mongolia cashmere goat, Liaoning cashmere goat, and Hexi cashmere goat, indicating differences in hair follicle activity between species. The depth of SF was smallest during the telogen stage, indicating that the fibers stop growing during this period. Although the density of the secondary follicle of the yak was not completely synchronous with its activity, the density was higher in the catagen stage than in the anagen stage, while its activity was lower, suggesting a dynamic change process in the hair follicles during the hair cycle.

### 4.2 Primary culture of fibroblasts derived from yak skin

Explant culture method is the prevailing technique for *in vitro* culturing of fibroblasts derived from animal skin. However, the selection of the age and source of the donor animal tissue requires consideration of species-specific characteristics and experimental requirements. Although younger donor animals are generally preferred for primary cell culture (58, 59), practical difficulties such as the thinness of fetal and neonatal yak skin require the use of digestive enzymes. Our study addressed this challenge by using thicker yak skin and employing a blunt dissection technique to reduce experimental time and costs. Although older donor animals were used in our approach, it remains essential to study animal aging (59), which offers distinct advantages to preserve wild or endangered species (60, 61), as age does not limit somatic cell nuclear transfer in older animals (62). We chose to source fibroblast cells from the abdominal skin of yaks, although ear tissue is conventionally favored for primary culture due to accessibility and minimal impact on animal functions (11, 63–65). However, evidence for superior fibroblast activity from sources other than ear tissue is lacking. Contrary to the prevailing assumptions, Luo et al. (66) found that abdominal fibroblasts of cattle outperformed ear and kidney sources in terms of cell culture and line establishment. In addition, fibroblasts derived from yak abdominal skin exhibited fewer mixed epithelial cell types compared to ear tissue, resulting in the elimination of further purification steps. Furthermore, the abdominal skin of the yak presented the added benefits of higher hair follicle density and a higher proportion of primary hair follicles, facilitating simultaneous isolation of high-quality dermal papilla cells.

Furthermore, we observed a relatively slow migration rate of fibroblasts derived from yak skin after the tissue fragment adherent method. On average, it took approximately 7 days for the fibroblasts to migrate, which aligns with the migration speed of the fibroblasts of various bovine breeds such as Luxi cattle (67), Swamp buffalo (66), and Sistani cattle (68), which required 7–14 days. However, previous studies using similar culture media reported migration times of 3–7 days for fibroblasts from horses (69), pigs (64), goats (70), and mice (11). These findings suggest the need to optimize the culture medium for bovine skin fibroblasts to improve their migration and aggregation speed.

### 4.3 Primary culture of subcutaneous yak preadipocytes

In the present study, we successfully isolated and cultured yak preadipocytes by enzyme digestion. Compared to the conventional tissue explant method, enzymatic digestion yielded higher efficiency and greater number of viable precursor cells (71), which was also a more cost-effective approach compared to the ceiling culture method (72). Given the high lipid content and dense ECM in ruminant adipose tissue (73), enzymatic digestion effectively releases cells by breaking down tissue structure, generating primary cultures of preadipocytes with high activity and proliferation. However, the deposition of lipids in grazing yaks exhibits considerable individual variations and seasonal fluctuations (74). To obtain optimal samples for cell culture, we collected subcutaneous fat from 1 to 2-year-old yaks during May to June when they were grazing on pasture. Notably, some previous research has shown that preadipocytes cultured using the ceiling method have a higher propensity for osteogenesis and adipogenesis, with higher proliferative activity (75). However, we observed significant differences in the culture microenvironment with the ceiling method. The ceiling method requires the use of an inverted culture bottle filled with culture medium to float the lipid-rich cells, creating a closed environment. Where adequate nutrient supply and low oxygen conditions may affect the growth characteristics of primary cells (76). Therefore, investigating enzymatic digestion to culture preadipocytes under these conditions could be an interesting research direction.

The differentiation of preadipocytes is a highly complex biological process regulated by multiple transcription factors. Among them, PPARγ and C/EBPα are the key transcription factors involved. For example, in the case of 3 T3-L1 cells, exposure to a mixture containing IBMX, dexamethasone, insulin, and rosiglitazone is commonly used to accelerate the expression of PPARγ and C/EBPα, promoting the formation of lipid droplets (43). However, the induction of preadipocyte differentiation may vary slightly between different species (8, 43). Fatty acids are considered essential for the differentiation of avian preadipocytes (77, 78), while in other species such as pigs (79), cows (80, 81), and goats (82), preadipocytes can be induced to differentiate *in vitro* by adding OA. In this study, we evaluated the ability of three methods, combining OA with the traditional cocktail approach, to induce the differentiation of preadipocytes in yaks. Our results showed that the addition of OA alone could induce yak preadipocyte differentiation, although with relatively low efficiency, as confirmed by the detection of PPARγ, C/EBPα, and Pref-1 during differentiation. However, the inclusion of OA in the traditional cocktail approach improved differentiation efficiency. Although our approach achieved successful results in terms of efficiency, determining the optimal dosage of additives remains a challenging task. As previously reported, high concentrations of OA can be toxic to preadipocytes (82), therefore achieving a standard protocol requires finding a balance between the dosage of exogenous additives, cell toxicity, and differentiation efficiency.

### 4.4 Primary culture of yak dermal papilla cells

Since the successful isolation of dermal papilla cells from mouse whiskers in 1981 (83) microdissection techniques have been widely recognized as the optimal method for obtaining dermal papilla cells. However, the practical operation of obtaining dermal papilla cells on a large scale still faces three key issues: (i) How to obtain more individual hair follicles from densely connected skin? (ii) How to efficiently flip the bulb region to facilitate the cutting and retrieval of dermal papillae? and (iii) How to ensure the secure attachment of the cut dermal papilla to the culture plate to enable feasible cell migration?

Research has shown significant effectiveness in using dispersing enzymes to isolate individual hair follicles from skin tissue (84). However, we encountered difficulties during the procedure, as the enzyme, while effective in separating epithelial tissue, tended to loosen the structure of the individual hair follicles, resulting in loss of follicular bulb. Consequently, we employed a physical approach to extract the follicular bulb. By longitudinally sectioning the skin tissue into a thinner plane, we were able to easily identify and obtain the hair follicles on the cut surface of the skin. For follicles enclosed by collagen fibers, we chose to utilize a spring clip to obtain the follicular bulb while trying to retain a longer outer root sheath tissue, to ensure that the pressure point of the Fine Tip Tweezers is distant from the dermal papilla during separation.

In the microdissection technique, the intricate task of turning the hair bulb inside out to expose the dermal papilla for cutting is extremely time consuming. We aimed to utilize digestive enzymes to loosen the structure of individual hair follicles to directly extract the dermal papilla from the base of the hair bulb. However, this approach required careful avoidance of dermal papilla digestion, which depended on the composition of the ECM of the dermal papilla. Although collagen type IV is present in the ECM of different species of dermal papilla, the content varies significantly, resulting in different phenotypes after digestion with collagenase IV (85–87). For example, collagenase IV does not fully digest the human dermal papilla (88), whereas it successfully produces a single cell suspension in the case of the dermal papilla of mice (89). Our study revealed that collagenase IV digestion effectively digested the hairy papilla of yaks, comparable to mice. Hence, precise control of the digestion time is crucial. We limited collagenase IV digestion of individual hair follicles to a maximum duration of 4 h, with the optimal timeframe being within 2–2.5 h. It should be noted that the composition and content of the ECM of the hair papilla exhibit variations throughout the hair cycle (88). However, our study specifically focused on yak hair papilla cell cultures during the anagen phase, making this method applicable only to this specific stage of hair growth.

Limbu et al. (11) described a technique that involved the enzymatic digestion of a single hair papilla to facilitate its adhesion in a Petri dish. Which is similar in principle to our technique. The difference is that we enzymatically digested the entire hair follicle containing the target hair papilla and then performed the separation of the hair papilla. However, this also allows one to obtain a dermal papilla that adheres quickly and stably, reducing both time and cost compared to other methods such as collagen coating, cell culture membrane coverage, or needle-based fixation (90). However, it should be noted that the critical step of transferring isolated dermal papilla to a new culture plate has not received sufficient attention. To address this, we recommend using gel loading tips and a single-channel pipette for transferring the dermal papillae, adjusting the equipment based on the number of papillae being transferred. It can reduce the risk of dermal papillae loss or damage during transfer.

## 5 Conclusion

This study utilized the Sacpic staining method to examine specific characteristics at different stages of yak hair follicles. During the telogen phase, the yak skin exhibited a loose arrangement of hair follicle clusters, resulting in easy detachment of the hair shafts and the absence of inner root sheaths. In contrast, during the anagen phase, the hair follicle clusters displayed a distinct structure, with a substantial increase in the primary and secondary hair follicles. During the catagen phase, the hair follicle clusters became incomplete, with a reduced number of follicles and shrinking of secondary hair follicles. Furthermore, the size, quantity, activity and depth of the secondary hair follicles were adjusted accordingly with the hair growth cycle, particularly during the anagen phase, when the secondary hair follicles exhibited their most vigorous development.

Furthermore, we successfully established primary cultures of fibroblasts, preadipocytes, and dermal papilla cells. All three cell types exhibited typical spindle-shaped morphology and exhibited an S-shaped growth curve, although their latent periods varied. Regarding the adipogenic differentiation of preadipocytes, we used three different induction methods, among which the modified cocktail method (DM3) achieved the fastest and most significant differentiation effect. Furthermore, by combining enzyme digestion and microdissection methods, we could more easily obtain hair papilla cells.

Taken together, the histological findings and improvements in cell culture techniques proposed herein provide a strong basis for future research on the intricate mechanisms underlying the growth of the yak hair follicle.

## Data availability statement

The raw data supporting the conclusions of this article will be made available by the authors, without undue reservation.

## Ethics statement

The animal study was approved by the Institutional Animal Care and Use Committee (IACUC) of the College of Veterinary Medicine of Gansu Agricultural University. The study was conducted in accordance with the local legislation and institutional requirements.

## Author contributions

BL: Conceptualization, Data curation, Formal analysis, Investigation, Methodology, Visualization, Writing – original draft, Writing – review & editing. YC: Conceptualization, Funding acquisition, Project administration, Resources, Supervision, Writing – review & editing. SY: Resources, Supervision, Writing – review & editing. JH: Project administration, Resources, Writing – review & editing. XY: Data curation, Methodology, Visualization, Writing – review & editing. SZ: Methodology, Formal analysis, Writing – review & editing. SL: Methodology, Resources, Writing – review & editing. PZ: Methodology, Resources, Writing – review & editing. HX: Methodology, Resources, Writing – review & editing. ML: Methodology, Writing – review & editing. XW: Methodology, Writing – review & editing.
