# Supplementary figures and images for: Histological characteristics of hair follicles at different hair cycle and in vitro modeling of hair follicle-associated cells of yak (Bos grunniens)
Source: Front Vet Sci. 2023 Nov 17;10:1277586. doi: 10.3389/fvets.2023.1277586 (PMC10691264; doi:10.3389/fvets.2023.1277586)

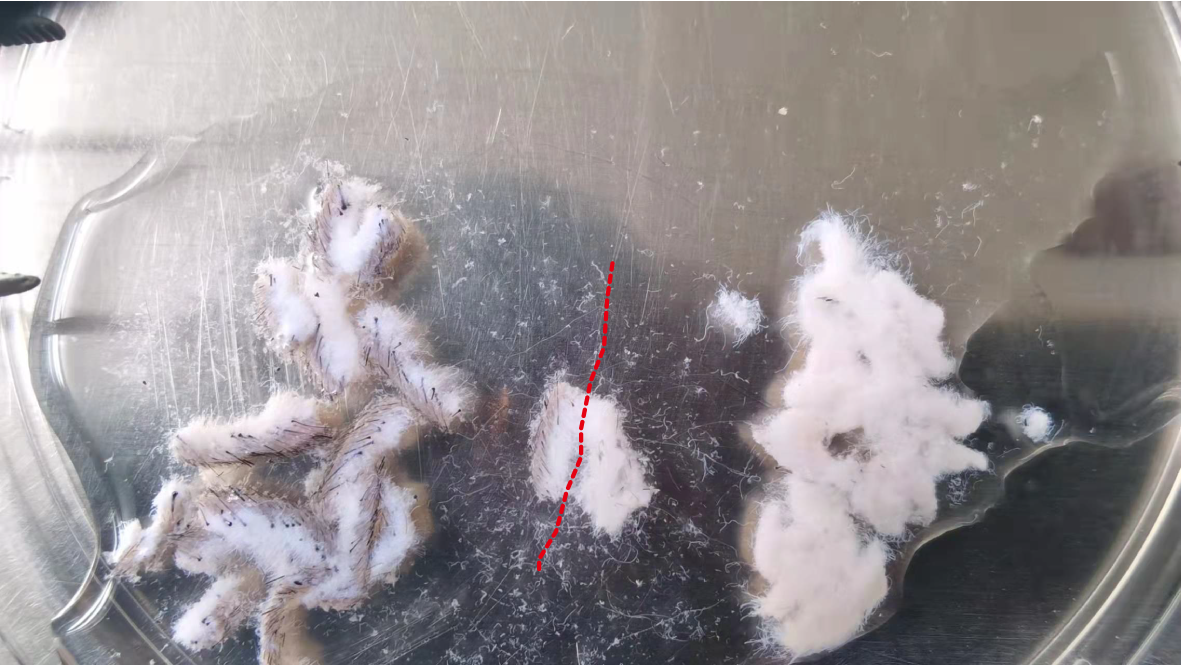

Supplement: Supplementary file 1 [file Image_1.tif]
